# Supplementary material for: Changing prevalence and factors associated with female genital mutilation in Ethiopia: Data from the 2000, 2005 and 2016 national demographic health surveys
Source: PLoS One. 2020 Sep 3;15(9):e0238495. doi: 10.1371/journal.pone.0238495 (PMC7470370; doi:10.1371/journal.pone.0238495)
Supplement: Attachment — Submitted filename: Response to Reviewers.docx [file pone.0238495.s002.docx]

Amy Michelle DeBaets, PhD
Academic Editor
PLOS ONE

Dear Dr DeBaets,

Once again, we would like to thank you for considering our manuscript and for arranging for it to be reviewed by reviewer(s). We have tried to address your comments and the comments / suggestions from the reviewer(s). In the ‘Response to Reviewers’, we copy each of the comments and provide the **RESPONSE** in bold underneath. We also provide a marked-up copy of the manuscript that highlights changes made to the original version and this is uploaded as a separate file called “Manuscript with Track Changes”. Finally, we provide an unmarked version of the revised manuscript without tracked changes and this is uploaded as a separate file labelled 'Manuscript'.

We hope we have satisfactorily addressed all the comments and hope that our paper may now be suitable for publication in your journal.

Best wishes

Gedion Asnake on behalf of the co-authors

**Academic Editor:**

While revising your submission, please upload your figure files to the Preflight Analysis and Conversion Engine (PACE) digital diagnostic tool, <https://pacev2.apexcovantage.com/>. PACE helps ensure that figures meet PLOS requirements. To use PACE, you must first register as a user. Registration is free. Then, login and navigate to the UPLOAD tab, where you will find detailed instructions on how to use the tool.

**RESPONSE:**

**We have uploaded our figure files to the Preflight Analysis and Conversion Engine (PACE)** <https://pacev2.apexcovantage.com/> **digital diagnostic tool. We have downloaded the converted figure files separately and resubmitted together with the revised version of the manuscript.**

**Reviewer #2:**

The authors responded to all of the concerns I raised in my initial review sufficiently. However, the abstract has not been updated to reflect changes to their methods and results: the prevalence estimate for 2016 does not correspond with what is reported later in the paper (line 195) and the methods still refer to regression adjustment for confounding. This must be corrected.

**RESPONSE:**

**Thank you for the encouraging response. We have updated the ‘Abstract’ section to reflect changes. On this ‘Abstract’ section, the prevalence estimate for 2016 (line 39) has been updated and now corresponds with what has been reported on the ‘Result’ section (line 195). We also modified the method section accordingly (line 36-38).**
